# All in the Family: Pets and Family Structure

**DOI:** 10.3390/populations1020008

**Published:** 2025-04-16

**Authors:** Leora E. Lawton

**Affiliations:** Berkeley Population Center, University of California, Berkeley, CA 94720, USA

**Keywords:** pets, families, children, demography, family structure, fertility

## Abstract

Although other studies have utilized demographic variables to characterize pet owners, this study incorporates a demographic framework that considers different family structures—couples and singles, with or without children—to better understand the place of pets within families. This article explores the extent to which pets fit into families in either substitute or complementary family roles, whether the inclusion of pets in families is subject to resource constraints or cultural proclivities. Data are from the 2023 Pew Research Social Trends study, a nationally representative telephone survey of 5073 respondents, analyzed using bivariate and multinomial models. Results indicate that couples without children are just as likely to be a dog-only household as are the traditional pet-owning families of couples with young children. They are also more likely to have cats, with or without dogs, compared to couples with young children. Homeownership makes pets, especially dogs, more feasible for families. The results suggest that pets are considered as substitutes and/or complements for other family members. Pets may be more affordable and attainable substitutes and/or complements for human family members, provided there are both human and spatial resources, filling a niche created by changes in family formation patterns, while providing affection, companionship and a sense of home.

## 1. Introduction

Nearly all pet owners—97%—in the United States see their pets as family members, and of those, about half see their pets as a family member just like humans [1–3], yet research in family sociology and demography has mostly considered them to be exogenous to the family unit, rather than part of the definition of family [4]. One reason for this exclusion may be epistemological: the study of humans and their pets is scattered across a wide range of disciplines, including, but not limited to, psychology, epidemiology, marketing, economics, social work, veterinary sciences and animal studies, as well as multidisciplinary journals. The ‘most important work’ to one scholar may be peripheral at best to another’s area of study. Another reason may be due to the well-founded conclusion among family sociologists and demographers that human beings are of critical importance for individual and family well-being, so the research focus has been on the many new human family forms emerging and being recognized in recent generations.

Pets have traditionally been seen in families with children as complements to the family unit. That pets might be replacements for children in a low-fertility society is increasingly a commonly heard refrain, yet household pets may also be replacing other family relationships, given other demographic changes in the family. Marriage, fertility and mortality rates are low in more developed countries, such that years of being unmarried and/or living alone have increased over the same time. Therefore, not only could pets be complements or replacements to children, but they could also be filling the roles of other absent household members, such as social companions and emotional support for those who, for example, lack partners or siblings. Although those researching pets have long considered this possibility and have provided some evidence in this regard [5], I have found no systematic comparative and quantitative study of different household structures in which pets are found. This article seeks to explore the relevance of pets in families and to bring family sociologists and demographers back into the discussion started decades ago [6,7] but largely disregarded since then. The objective of this research is to understand the contexts where pets fit into families. I begin with background about pets in United States society, followed by a review of demographic theory and its connection to the family pet. The statistical analysis of pets in families is followed by a discussion of these patterns and the demographic transition and implications they hold.

The increasing ubiquity of dogs in public spaces—streets, dog parks, grocery stores and even restaurants—suggests that the societal meaning of pets is evolving. Signs of the rising prioritization of pets in social life abound. One example is the popularity of cat cafes, which are coffee shops where customers can interact with cats and perhaps adopt them. The first of these cafes was opened in Taiwan in 1998; they then spread to Japan and are now popular in other Asian countries, the United Kingdom and North America [8]. Pet accessories now go beyond collars and food dishes: discriminating consumers can purchase upscale cat furnishings, where “cat towers, litter boxes and other pet accessories are given a designer treatment in order to harmonize better with tasteful home design” [9] (p. J20). Pets are increasingly being accepted for burial in human cemeteries [10]. Some pet owners insist that people who have pets are not owners but are guardians. A department store, JC Penney, advertised photo sessions for the family cat. The boundaries for pets versus humans have blurred.

The shift in pets from simply companion animals to non-human family members is also reflected in tracking sales of items for pets. In 2016, pet food sales in the United States were at $28.2 billion and supplies at $14.7 billion [11], and in 2022, annual sales of pet food and treats had more than doubled to $58.1 billion [12]. Over half of pet owners buy their pets gifts for the holidays [13], and two-thirds mention their pets in holiday greeting cards [14]. The share of discretionary spending on pets annually, about 1% for households on average, is more than annual spending on alcohol, phone bills or men’s and boys’ clothing. Increasingly, pets are covered by health insurance, given their valuable position in the family, reaching $3.2 billion in premiums in 2022, up from $774 million in 2016, with the number of insured pets in the United States increasing from 1.6 million in 2016 to nearly 4 million in 2022 [15]. While pet expenditures pale in comparison to what is spent on children by direct family consumption, let alone public spending on healthcare and education, they also provide evidence that pets have an increasingly prominent place in homes.

### 1.1. Pet Owning Households

Accessing accurate data for research on pets is not straightforward. Different organizations collect data for different purposes and use different methodologies for sampling and inclusion, which makes comparability, reliability and validity questionable [16], something that was a concern even during the ‘golden age’ of telephone and face-to-face probability surveys and now even more relevant in the days of typically opt-in internet-based data. Two industry groups are the most frequently cited for national totals: the American Pet Products Association (APPA) and American Veterinarian Medicine Association (AVMA), but neither releases microdata for public use, and membership is restricted to relevant industries and professions. Both currently use opt-in online panels and ‘balance’ the sample to approximate the overall US population, but the sample itself is not representative. For example, 15% of Americans do not have smart phones [17], and one in five do not have broadband access at home [18]. Their estimates have also diverged since the 1980s, such that “APPA estimates are now about 20–25% higher than the AVMA estimates” [19] (p. 1). Two other market data sources are Euromonitor International and Packaged Facts International, which allow for international comparisons.

Given those caveats about data quality, estimates for the number of pets from several sources over the past 40 years are presented in Table 1, displaying a wide amount of fluctuation due to the different data collection methodologies. The three estimates presented for 2012 from AVMA, APPA and Euromonitor are noticeably different. Despite the variation from source to source, data consistently show that fewer households have cats than dogs, but there are more multi-cat homes than multi-dog homes. According to the APPA 2022 National Pet Owners Survey, 39 and 33 percent of U.S. households own at least one dog or cat, respectively. Although precise numbers are not available, it is nevertheless clear that pets are prevalent throughout much of American society.

### 1.2. Animal Demography and Households

Beyond the increase in numbers for the US pet population seen in Table 1, other demographic trends suggest the diffusion of pets into different types of families. If the primary motivation for pets was for the children, one might expect that as the number of married couples plus school-age children households declines in a low-fertility and aging population, the number of pet-owning households would also fall. In 1960, 51.8 percent of American households were married couples with children under 18, declining to 33.7 percent in 2005 and then to 27.8 percent in 2022. In 1960, 13.1 percent of households consisted of one person, increasing to 26.6 percent in 2005 and then to 29 percent in 2022 [20,21]. Likewise, the percentage of family households without children under 18 has increased in the past generation. In 2020, 40% of all families lived with their own children under 18, down from 44% in 2010 and 48% in 2000 [22]. Yet, as seen in Table 1, the average number of cats and dogs per household fluctuates, undoubtedly due at least in part to the data collection processes, but the changes are not as dramatic as those to household composition. Rather, the total number of pets has increased even as the proportion of households with children has declined. Therefore, either the traditional pet-owning households have more pets per household than before, or pets are increasingly present in households without children. Older ‘empty nest’ households are increasing in proportion as well, and although this cohort is associated with declining pet ownership in older ages, the pattern may be shifting. At the same time, the younger cohort may be acquiring pets at an earlier phase in their life course, prior to marriage and childbearing, due to the higher ages for those two life events [23].

### 1.3. Human Demography and Pets

Traditionally for the United States in the twentieth century, parents would acquire a pet when the children were old enough to clamor for one [7,24,25]. Households with children or adolescents also had higher rates of pet ownership because parents think pets will teach and comfort children, and because there is a sense that the home will be stable enough to support a pet [26]. Albert and Bulcroft [7] found that pet acquisition and attitudes towards animals were seen to vary across seven stages of family development, from the pre-married stage through the different age ranges of children, post-parental or ‘empty nest’ phase and finally widowhood, where acquisition increases to a peak during the child-raising years and declines thereafter. Using a multivariate analysis, their results showed that families with school-age children, parents in their mid-forties and mid-fifties and remarried parents were the most likely to have pets, whereas pet ownership was low among widows, empty-nesters and families with infants. A similar result using a bivariate analysis was found in subsequent years by Poresky and Daniels [27] and Maier [28].

Pets have long existed in households without children [29], but it has become popular in recent years to state that one of the reasons that pets exist in households without children is because they are increasingly a substitute to having children in a low-fertility society [30,31]. Anecdotally, one hears that pets are like children in statements such as, “Since we don’t have children, the dog is the joy of our life” (San Diego passerby, 2 May 2015), a sentiment documented in qualitative research [32–34]. The idea that pets are replacements for humans is a discussion that would not have happened prior to the demographic transition. With people having fewer children and, when doing so, at older ages, pets have increasingly become companions and providers of social support, such that “interaction with companion animals may not only supplement human companionship, but may actually replace it” [6] (p. 11). Given that the cost of having and raising a child in developed countries is high—likened to owing a Ferrari, the ultimate luxury good [35]—it may be that the more affordable object of emotional attachment for some individuals or couples is a dog or cat, if they have been priced out or have opted out of a higher parity or parenthood altogether. Demographers have shown how the number of children is a function of economic status, such that, in a more-developed economy, the cost of raising children can dampen fertility, as does the opportunity cost for lost wages for mothers [36–38]; see Morgan and Taylor [38] for review. When childcare, housing and educational costs rise, an additional child, or any child, may be unattainable for those without resources.

Yet, there are other motivations for pets. Even though pet ownership is higher among those with a child present in the home than those with two or more children, attachment to pets is lower compared to those in households with one child and in couples outside of the ‘married plus children’ phase [7]. Similarly, Poresky and Daniels [27] found that attachment was higher for divorced compared to married persons, and there was a curvilinear pattern for age, with it being highest for the middle aged. In addition, pets meet different emotional needs during these phases [6], as companions for either or both the adults and children, depending on one’s position in their family of orientation or volition.

Beyond the motivation for pets in general, there is the fact that dogs and cats are different species with different needs and behaviors, which could determine their placement in families. Dogs and cats require different kinds of care; notably, dogs require multiple daily walks, whereas cats can be left alone in the house for longer periods of time. Perhaps for this reason alone, it may be logistically easier to raise a dog with two adults in the household, whereas a cat requires a more flexible set of tasks. Placing pets within a physical context is also important to consider. Dogs tend to need more space than do cats, which makes a house with a yard attractive, and landlords are less likely to rent to dog owners than to cat owners [39]. Accordingly, housing type may also be salient for having pets, and specifically dogs versus cats. Rural dwellers have space for pets compared to urban dwellers, and dogs are desirable in more rural environments. In contrast to dogs, there is growing popular acceptance that cats are better off living indoors, so there may not be a strong effect of homeownership on having a pet cat.

There are also cultural predispositions to having household pets. Asian and Hispanic families predominant in the United States tend to come from societies where pets are less common. While many countries are typified by pet ownership, it is a phenomenon more pronounced in the United States than in most of Latin America [29], China and South Asia [40]. Black families have had a mixed relationship with pets, particularly dogs. Dogs had been used to control enslaved people, which left a lingering cultural wound [41]. At the same time, dogs are useful for home security, particularly in less-affluent neighborhoods. Black Americans are more likely to be renters, however, so there is less opportunity to develop pet-owning behavior, as having had pets in the home as a child is a salient predictor for pets in the home as an adult.

Understanding the impact of demographic processes and their relationship to family structure [42] can provide insight into the presence of pets in the family. By 2022, nearly all developed countries reached below-replacement fertility, meaning that people have fewer children, fewer people have any children, those who do have them later in life, and more households consist of no children. The ‘marital transition’ consists of a higher age at first marriage for men and women relative to the mid-twentieth century, having risen for both men and women from the age of the early twenties in 1960 to the late twenties in 2016 [43], which in turn leads to more early adult years outside of marriage [23]. The decline in the number of children means fewer years as a child-centered family. Higher rates of never marriage, divorce and widowhood, coupled with a long life expectancy and a decline in norms for coresidence [44], mean that a higher proportion of adults live in households either with only a spouse or with no other family member [45]. For these reasons, pets could fill the gap formerly met by humans, including spouses [46].

In addition to these large demographic changes in family formation, definitions of the family itself have expanded. Families are now recognized to consist of a wide range of forms, including blended families with half- and step-relatives, single-parent households, including those headed by fathers, and same-sex couples with or without children, as well as ‘like family’ close friends [47]. These expansive definitions of family are particularly important for those living outside of married or consensual couplehood. With this expansive definition of family members, it becomes compelling to consider what families are and what they do for each other. Family demographers argue that a healthy two-parent household is the best place for raising children, yet other family practices are essential as well [38]. Beyond the conjugal relationship, spouses provide emotional, financial and instrumental support, companionship and friendship. Children provide their parents with a meaning to life and a means to create personal networks within communities, which themselves are a form of social support. Adult children may also provide companionship and, in later life, often take on the role of caregiving for aging parents. Other family members step in to provide many of these social and instrumental support roles. All family members are potentially a source of support in times of short-term crisis or long-term dependency. Good friends provide some of these forms of support, though typically do not provide hands-on physical care or management of finances.

In this mix of fluid family forms is the family pet, where the meaning of family accommodates having pets as the non-human yet participating family members. In addition to the instrumental roles of teaching children responsibility, home security or rodent control, pets may provide companionship, emotional support and a reason to exist, or, in other words, some of the same roles as those performed by humans. As such, including them as family members is a logical step, and the breadth of support roles filled by pets suggests that their presence would not be primarily for, or like, a child [46,48].

Qualitative studies have documented the expanded family relationship quality viewed by people for their pets.

“Marx might be a dog, but he’s like my child—a human being. Like a kid—he needs to be taken care of. He’s just like a member of the family. Our lives revolve around him” [49] (p. 424).

“All through my childhood he [the dog] was there as companion and comforter. I was an only child, and so I regarded him as my brother” [48] (p. 721).

“Sadly, my husband died . . . They [two pet dogs] were an absolute blessing for me, especially Sarah who sensed my sadness, and for the first three months I was on my own, slept on my bed every night. I had a very close relationship with Sarah, which lasted for 12+ years and she left a big gap in my life when she died” [48] (p. 722).

Clearly for some, dogs and cats are far more than just pets, exogenous to the definition of family, and fill the space where humans are lacking.

### 1.4. Hypotheses

The analysis will address the following hypotheses regarding pets and their presence in families:

- Given that fertility levels are low and even declining, pets are substitute as well as complementary goods for children, so couples without children will be as likely to have pets as those with children.
- Given the expanded range of time spent outside of marriage for Americans, pets can fill the void when there is no partner, so pets, particularly cats, who may be less labor-intensive than dogs, will be in households without a spouse as much as in households consisting of couples plus children.
- Acquiring a pet is contextualized by housing, so it is expected that households who live in owned, rather than rented, homes will be more likely to have pets, and rural and suburban residents will be more likely to have pets than urban dwellers.
- Given the different cultural patterns underlying pets in the household, pets will be less common in families where the respondent is not native-born in the United States and in non-white or Hispanic families.

A major contribution of this paper is to systematically explore the kin-like positioning of pets within distinctive family structures, using a multivariate quantitative analysis and a population-based sample.

## 2. Materials and Methods

### 2.1. Data

The data utilized for this study were from the publicly available Social Trends study by Pew Research [50]. The sample was drawn from the nationally representative American Trends Panel. Conducted by telephone, it consists of adults aged 18 and older, living in the continental United States, with n = 5073 (Margin of Sampling = 1.7% for weighted data), with a response rate of 87% from its proprietary American Trends panel. It contains the rich data regarding pet ownership, attitudes about pets and family structure characteristics necessary for this analysis.

The dependent variable for pets is based on a set of questions: “Do you currently own any pets?” and if answering yes, then, “Do you own any cats or dogs?”, with the answers being only cats, only dogs or both cats and dogs [51] (pp. 12–13). These variables were used to construct the dependent variable consisting of four categories: does not own a dog or cat, owns only one or more cats, owns only one or more dogs and owns both cat(s) and dog(s). This variable excluded 116 owners of pets other than cats and dogs, or 3.7% of the pet-owning sample.

The primary independent variable consisted of six family structures, using marital status and whether the respondent has children 18 years old and older or 17 years old and younger, regardless of whether they live in the home. Even after the children leave the nest, the presence of a pet may be a holdover from the childhood pet, or parents found that they liked having a pet in the house even after the children were not. The never-married, divorced, separated and widowed were combined into ‘single’, because there were few never-married older respondents and very few formerly married younger respondents. Similarly, partnered respondents were combined with married due to small cell sizes. The six family structures are married or partnered, no children, married or partnered with children <18, married or partnered with children 18+ only, single no children, single with children <18 and single with children over 18 only.

Excluded were those living in ‘other’ housing arrangements. While homeowners could be living in condominiums and thus lack yards, a cross-tabulation of the data for homeownership and dwelling type in the General Social Survey (DWELOWN and DWELLING) indicated that 85% of homeowners are in single-family homes, compared to 30% of renters, although since 2014, that has declined to about 79% (author analysis). Also controlled was residence location, with dummy variables for urban and rural and with suburban as the reference category.

Additional independent variables were included to examine the cultural proclivities of pet ownership as reflected in dummy variables for race and ethnicity (Black non-Hispanic, Asian and Hispanic, with White non-Hispanic as reference). Immigrant status is represented in a dummy variable for whether one is a native-born American. Some Asian populations, particularly Japanese, have a long history of pets, especially cats, but the category of Asians in the United States is so diverse ethnically that it is difficult to provide hypotheses for Asian Americans and pet ownership with these data. Despite that, a dummy variable for Asian is included. Socioeconomic controls included income (a categorical variable for income groups, with missings and refusals recoded to the average and a dummy variable for missings) and whether one has a college degree. The variable for age is an ordinal variable, with four age groups: 18–29, 30–49, 50–64 and 65 and over. Gender was also controlled (male, female reference; there were no missing responses). Descriptive characteristics for these variables can be found in Table 2. The total number of cases in the multivariate models after removal of missing cases was 4841.

### 2.2. Methodology

The first set of analyses are bivariate, comparing family household structures and pet ownership. Following these initial analyses, the primary analytic models for the individual-level data are multinomial (that is, polytomous) regression models to test predicted probabilities for pet ownership statuses—cat owners, dog owners, both cats and dogs—compared to having neither of these pets, using the contextual and control variables as described above. Data are weighted except where noted.

## 3. Results

### 3.1. Descriptive Statistics

Pet ownership for each of the sample characteristics is presented in Table 2. On average, about 60% of respondents have pets, with more dog owners than cat owners, with substantial variation between subgroups. This table separates the partnered from the married respondents in order to show greater detail, whereas they are combined in the multivariate analyses due to cell sizes. While past research found pet ownership to be most common among families with young children, these data show that married respondents either without children or with grown children are just as likely to have pets. Similarly, partnered respondents with or without children are just about as likely to have any pet, providing initial support for Hypothesis 1, that they exist both as replacements for non-existing children and complements in households with children. A lower percentage of single adults own any pet compared to married respondents, and about twice as many have any pet compared to single adults with no children. Another sign of pets as replacements is that pet ownership is prevalent throughout all age groups and only declines in older ages for dog owners, perhaps given the amount of daily and active attention they require. In contrast, the ownership of cats only remains relatively stable across the life course. Even at age 65 years and older, nearly one-half of households have a pet, compared to 63% for ages 30–49. The challenges of cohort versus period versus age effects certainly exist here, but one can conclude that even though prevalence of pets drops off in older ages, it remains common. Just under half of renters have no pets, versus about one-third of homeowners, and the difference is largely due to fewer dogs. As for native-born Americans, about half have pets compared to one-third of those born outside the US, and in this case, the difference is largely due to fewer cats. Finally, the results for race and ethnicity show that 70% of Blacks have no pets, twice as many as White non-Hispanic and Hispanic respondents.

### 3.2. Multinomial Model

The multinomial regression analysis is presented in Table 3 and provides odds ratios for pet ownership: cats only, dogs only, both cats and dogs, compared to the reference category of those with no cats or dogs. The primary explanatory variables in the model are the five family statuses compared to the reference category of the traditional family structure for pets, that is, married with children under 18.

**Hypothesis 1** (children and/or pets). *The results show that in contrast to previous research on pet ownership, married or partnered households with no children are actually more likely to have cats with or without dogs (OR = 1.390 and 1.715, respectively) than married or partnered with young children, once all other factors are taken into consideration. Further, they are just as likely to have dogs without cats.*

**Hypothesis 2** (pets without partners). *As hypothesized, singles with no children are about half as likely to have any dogs with or without cats (OR = 0.452 and 0.537, respectively) compared to married/partnered with young children. However, single adults with children are no different than married couples with children for the three pet categories.*

**Hypothesis 3** (physical setting). *Homeownership is an important factor in the ability to have a pet. For all combinations of pets, being a homeowner versus a renter made it much more likely to have a pet, ranging from 36% more for just cats to over twice as likely for those who have both dogs and cats. Geographic setting is also important: as we expected, rural residents are more likely to have pets, especially both cats and dogs (OR = 1.747), compared to urban residents, and urban residents have fewer dogs compared to suburban residents.*

**Hypothesis 4** (race and ethnicity). *Those born outside the United States were hypothesized to be less likely to have pets, and that is the result. The native-born were nearly two times as likely to have cats alone (OR = 1.889) and about one third more likely to have dogs with cats (OR = 1.413) or without cats (OR = 1.342). As for race, Blacks are considerably less likely to be any kind of pet owners compared to Whites, especially owners of cats (OR = 0.142 for just cats, 0.117 for both cats and dogs), whereas Hispanics are less likely to have cats in the household compared to non-Hispanics, but there is no difference with dogs. Asians are considerably less likely to have any pets (OR = 0.430, 0.352 and 0.275, respectively, for cats, dogs and both).*

Other results are consistent with previous research. The older one is, the less likely one is to have any pets. As for two measures of socioeconomic status, income is not significant, and education interestingly decreases the likelihood for having both cats and dog, by over half (OR = 0.431) for those who have both cats and dogs and by a quarter (OR = 0.758) for dogs alone. Finally, men are about 25–40% less likely to report having any pet.

## 4. Discussion

The results of this analysis provide support for all four hypotheses. First, the result that pets are even more likely in couples without children under 18, compared to those with, suggests that pets can ‘replace’ children in the former, while retaining their role as complementary goods in the latter. Second, dogs are more likely to be in households of couples, perhaps because, as with children, it is easier to manage a pet with a partner. Cat ownership is more likely in childless married/partnered households compared to those with children, possibly because children, or their friends, may be allergic. Singles with or without children are as likely to have cats as couples with children, possibly because it is easier to have a cat than a dog without a partner because cats require less hands-on care.

Third, there are also settings where pets are more likely. Homeowners are more than twice as likely to have dogs and a third more likely to have cats compared to renters, demonstrating the importance of physical resources for being able to add a pet to the household for several reasons. Renters are often unable to find housing allowing pets, and when allowed, the pet is more likely to be a cat, as they do not bark and disturb neighbors [39]. Owned homes tend to be single family houses, and there is a selectivity effect in that people who want or have dogs purposely select a home with a yard. Relatedly, some adoption organizations require a fenced yard as a condition for acquiring a dog [52]. Further evidence for the physical context is provided by the fact that urban dwellers are less likely to have dogs, while rural residents are the most likely. Thus, dog ownership, and to a lesser degree cat ownership, is partly structured by the ability to find or afford suitable housing. Finally, for the fourth hypothesis that cultural norms play into pet ownership, as evidenced by the results for race, ethnicity and immigrant status, the results showed that native-born White Americans are more likely to have pets than Black or Asian households, with the exception of Hispanic households, who are just as likely to have dogs as non-Hispanic households. The dominant immigrant populations flowing to the US from South America and Asia have not been pet cultures, although the presence of household pets is increasing [40]. As economic factors are controlled, the results point to a cultural explanation, such that pets are not necessarily an expectation for inclusion in a family household.

All told, these results indicate that pets fit into families when the families have the motivation for a pet, when the living space is suitable or permissible for pets, and when pet owners are able and willing to take on the responsibility of pets. The multivariate model allowed a detailed drill-down into family structures previously not seen, revealing that married and partnered couples with or without children are households where pets are well established, in contrast to the research from the twentieth century, where the combination of marriage and having school-age children is the most important predictor for pet ownership [7]. The importance of family structure is revealed when examining different structures separately, not just married versus not married and children versus no children; rather, creating combinations of those different dichotomies sheds more light on why someone might have pets. Moreover, the kind of pet is also an important factor in ownership patterns, in part likely due to the burden of care and in part due to residential location and setting.

The results here align with Bouma et al.’s analysis of cats in the family [5], pointing here to four niches for pets depending on family structure: complements or substitutes for children and complements or substitutes for adult companions. For each family type, both complements and substitutes may be at work in that dogs are seen in households of couples with or without children and cats seen in households without spouses or children about as much as in households with couples. Reasons for either substitution or complementary presence of pets are multi-faceted [53]. Economic factors such as homeownership may directly and indirectly affect the decision to acquire a pet instead of or in addition to a child. Unlike with children, pet acquisition has a low economic opportunity cost because it typically does not interfere with or complicate schooling or career development. Normative and emotional reasoning also may be at work. It is an easier decision to acquire a pet than it is to have a baby, and the consequences of an unplanned pet are typically less encompassing than an unplanned child. With the later ages of marriage and first birth, the idea that singles and couples acquire a pet in the interim or as ‘practice’ may be compelling. It is also easier to find a suitable pet to share a life with than a romantic partner. People speak of their ‘fur babies’ but not ‘fur spouses,’ instead calling the pet a ‘best friend’ or ‘family member’. While it is not possible to discern from this analysis whether pets are an inferior good or preferred choice, or whether a pet is a rational choice for others who have space in their families unfilled by humans, pets may be able to at least partially fill the void left by the absence of a spouse and/or children. In other words, pets have increasingly become, not companions for children, but companions for childless couples, and pets, particularly cats, are increasingly companions for the unpartnered.

The broadening role of pets has further demographic implications. Consider that most elder care—physical, financial, social—is provided by spouses, followed by adult children, and is care that pets cannot provide, even as they provide valuable companionship. Moreover, this role for pets may yet expand as economic trends appear to further dampen fertility: an increasing proportion of young adults live in their parental home, and of those, 25% neither work nor go to school. Real wages of young men aged 25–35 have declined since 1980 [54]. It is therefore likely that these secular changes in society are altering expectations for family and household formation, such that living with humans is less normative, whereas living with a pet is more prominent in American culture. Much of the same logic for when and how to become a pet owner aligns neatly with many of the same motivations for not becoming a parent, e.g., “When you’ve got children, you have got to sacrifice so much and I’m not prepared to do it. It means that I am me, not someone’s mother. It means having freedom” [55] (p. 48). At the same time, owners of dogs and cats are frequently encouraged to consider pets like children. As fewer and fewer people have children, familiarity with the joys of parenthood—as opposed to the well-advertised sacrifices and headaches–fades, making parenthood even less likely as a normative expectation. Normative pressures for childbearing (as opposed to pet ownership) accumulate as peers forego parenthood. With parenthood out of reach or perhaps out of vogue for more than one generation, people may cease to fully appreciate that their ‘fur babies’ are quite distinctive from human children and far more limited.

Pets increasingly fill a niche for a less-expensive and more-attainable relationship than children in which to expend disposable time and resources for affection, companionship and a sense of home. Future research on pets is needed to understand the relationship between pets and fertility patterns and decisions and the effects of pet ownership on physical, emotional and social well-being. New data will be necessary to both disentangle the age, period and cohort effects and link topics of research previously considered unrelated. Comparisons with other countries with different norms, housing and demographic characteristics would add insight. Low fertility alters the societal fabric, and one consequence includes alternate family forms, as well as the overall reduction in the salience of parenthood. As such, pets could be a reasonable coping mechanism or substitute in order to transition to a sustainable population size.

### Limitations

There are certain features of the Pew data that limit interpretation. The variables measuring whether the respondent has children do not distinguish between those children who live in the home and those who do not. Some of the married couples might not have had children residing in the home full-time, which might make them more like couples without children, which would reduce the difference between the two groups. These 2023 survey data did not include sexual orientation, where pets may be children for lesbian and gay couples. The data are from after the COVID-19 pandemic, which may have led to at least a temporary increase in pet adoptions [56] by households that might not have considered pets previously. The pandemic also led to a mixed-fertility response of a decline among foreign-born women and increase among native, college-educated women [57]; such macro-effects could motivate a decision to acquire a pet. Cross-sectional data cannot distinguish between cohort, period and age effects. Nor do we know the motivations behind decisions to acquire pets or to remain pet-less. That said, the data demonstrate that pets fit into a wider range of family structures than previously documented.

## 5. Conclusions

Contemporary research on the family has focused on new forms of families and changes in marriage patterns, and yet here is a change in household structure that has gone under the radar for many family scholars, even though over half of Americans have pets, and nearly all pet-owning Americans say the pet is part of their family. The conclusion for this exploration into pets in the family aligns with Seltzer’s recommendations for family demographers who, in her essay on changing family forms, need to consider both “what family means, how families are organized”, as well as “what makes family relationships unique” [58] (p. 421). The integration of pets in the family speaks to emerging forms of family structure and to the salience of family in the 21st century, or rather, to how people find affection and support and how they do not.

## Figures and Tables

**Table 1.** Trends in dog and cat presence in households (HH).

	1981 *	1991 *	2001 **	2006 ***	2012 ***	2012 **	2012 +	2020 ***	2024 +
Number of Dogs	53,831,000	53,272,000	61,293,000	72,100,000	69,900,000	71,168,000	83,000,000	85,582,105	89,700,000
Number of Cats	44,579,000	62,434,000	75,601,000	81,700,000	74,100,000	73,394,200	95,600,000	61,542,654	73,800,000
Total Number of HHs in US †	82,638,000	94,312,000	108,289,000	114,384,000	121,084,000	121,024,000	121,084,000	128,451,000	132,216,000
Average Number of dogs/HH	0.651	0.565	0.566	0.630	0.577	0.588	0.685	0.666	0.678
Average Number of cats/HH	0.539	0.662	0.698	0.714	0.612	0.606	0.790	0.479	0.558

Source:

* Packaged Facts International 2014

** Euromonitor International

*** AVMA

† US Census household counts, Table HH-1, November 2022.

+ APPA.

**Table 2.** Percentage with pets by groups.

	No Dogs or Cats	Cats	Dogs	Dogs & Cats	Number
**Total**	40.4	14.0	30.4	15.2	5059

**Family Structures**					
Married no children	36.2	17.3	30.6	15.9	759
Partnered no children	27.2	21.1	28.9	22.8 ^1^	276
Married children < 18	35.7	10.6	35.5	18.2	977
Partnered children < 18	23.3	18.0	37.0	21.7 ^1^	156
Married children 18+ only	38.8	12.0	35.0	14.2	980
Partnered children 18+ only	27.8	6.7 ^1^	42.2 ^1^	23.3 ^1^	76
Single no children	51.5	15.4	23.4	9.7	1022
Single children < 18	42.0	10.7	30.3	17.0	307
Single children 18+	50.0	14.7	24.5	10.8	455

**Socioeconomic Variables**					
Home renter	48.7	13.9	25.8	11.7	1442
Homeowner	36.5	14.0	32.6	16.9	3545
Has Bachelor’s+	44.5	15.5	30.0	10.0	2189
Less than BA/BS	38.2	13.3	30.7	17.9	2868
White non-Hispanic	33.9	16.8	31.7	17.7	2717
Black non-Hispanic	70.3	5.0	20.6	4.1	803
Hispanic	36.6	10.5	37.1	15.8	954
Asian	65.9	10.1	17.4	6.5	185
Native-born American	38.4	15.2	30.8	15.6	4441
Not native-born American	49.8	8.2	28.9	13.1 ^1^	940
Male	44.4	13.0	29.3	13.2	2673
Female	36.7	14.9	31.8	16.6	2336
Urban	50.5	13.2	25.8	10.5	1420
Rural	30.2	15.1	32.1	22.6	1121
Suburban	40.4	13.8	31.9	13.9	2509
Age 18–29	38.5	15.3	29.8	16.3	660
Age 30–49	37.0	13.4	31.5	18.1	1791
Age 50–64	38.6	12.3	33.5	15.6	1402
Age 65+	48.9	15.9	25.7	9.5	1195
Average income group	4.69	5.20	4.86	4.76	5073

1 Fewer than 30 cases in group. Note: All percentages and means weighted; counts are unweighted. All tables have chi-square tests significant at *p* < 0.001.

**Table 3.** Multinomial logistic regression models of pet ownership ^a^.

	Cats Only			Dogs Only			Both Cats and Dogs		

Explanatory Variables	B	Odds Ratio	SE	B	Odds Ratio	SE	B	Odds Ratio	SE
Married or partnered, no children ^b^	0.539 **	1.715	0.151	0.072	1.075	0.120	0.330 *	1.390	0.144
Married or partnered, children 18+	0.054	1.056	0.174	0.194	1.215	0.128	0.097	1.102	0.163
Single, no children	*−*0.045	0.956	0.154	*−*0.622 **	0.537	0.122	*−*0.794 **	0.452	0.159
Single, children < 18	*−*0.193	0.824	0.236	*−*0.107	0.898	0.165	*−*0.175	0.839	0.206
Single, children 18+	0.077	1.080	0.202	*−*0.287	0.750	0.161	*−*0.364	0.695	0.212
Urban (suburban ref)	*−*0.037	0.964	0.119	*−*0.248 **	0.780	0.094	*−*0.333 **	0.717	0.130
Rural (suburban ref)	0.230 *	1.259	0.117	0.216 *	1.241	0.093	0.558 **	1.747	0.111
Homeowner (renter = ref)	0.312 **	1.366	0.116	0.498 **	1.645	0.091	0.744 **	2.104	0.119
Income categories	*−*0.024	0.976	0.019	0.022	1.022	0.015	*−*0.021	0.979	0.019
Income missing	*−*0.787 **	0.455	0.284	*−*0.176	0.839	0.187	*−*0.208	0.812	0.239
College (less than BA ref)	*−*0.064	0.938	0.107	*−*0.277 **	0.758	0.086	*−*0.842 **	0.431	0.116
US native (non-native ref)	0.636 **	1.889	0.162	0.294 **	1.342	0.113	0.346 *	1.413	0.151
Black non-Hispanic (White non-Hispanic ref)	*−*1.953 **	0.142	0.215	*−*1.029 **	0.357	0.123	*−*2.143 **	0.117	0.230
Asian (White non-Hispanic ref)	*−*0.843 **	0.430	0.245	*−*1.043 **	0.352	0.191	*−*1.290 **	0.275	0.285
Hispanic (White non-Hispanic ref)	*−*0.373 *	0.689	0.156	0.166	1.181	0.114	*−*0.243	0.784	0.147
Male	*−*0.419 **	0.658	0.096	*−*0.324 **	0.724	0.075	*−*0.496 **	0.609	0.097
Age 18–29 (age 30–49 ref)	*−*0.092	0.912	0.146	*−*0.307 **	0.736	0.117	*−*0.211	0.809	0.143
Age 50–64 (age 30–49 ref)	0.365 *	1.440	0.144	0.198	1.219	0.111	0.553 **	1.738	0.140
Age 65+ (age 30–49 ref)	0.587 **	1.798	0.151	0.817 **	2.264	0.124	1.502 **	4.491	0.163
Intercept	*−*1.911 **		0.381	*−*0.936 **		0.295	*−*2.285 **		0.374

a The reference category for the multinomial model is no cats or dogs.

b The reference category for the family statuses is married/partnered with children <18 in the household.

** Significant at *p ≤* 0.01

* *p ≤* 0.05. Note: Data are weighted.

## Data Availability

The original contributions presented in this study are included in the article. Further inquiries can be directed to the corresponding author.
